# Early maladaptive schemas in adolescents with attention deficit hyperactivity disorder

**DOI:** 10.3389/fpsyt.2024.1455897

**Published:** 2024-10-04

**Authors:** Ozlem Sireli, Mehmet Colak, Tugce Hilda Demirci, Ayse Ece Savascihabes, Hatice Oz Cinar

**Affiliations:** ^1^ Department of Child and Adolescent Psychiatry, Sivas Cumhuriyet University Faculty of Medicine, Sivas, Türkiye; ^2^ Department of Child and Adolescent Psychiatry, Freelance Physician, Izmir, Türkiye

**Keywords:** ADHD, adolescent, early maladaptive schemas, psychopathology, child

## Abstract

**Introduction:**

Current evidence suggests that early maladaptive schemas are affected in attention deficit hyperactivity disorder (ADHD). Most of the studies on the subject have been conducted with adults, but the number of studies conducted with adolescents with ADHD is quite limited. The aim of this study was to evaluate early maladaptive schemas in adolescents diagnosed with ADHD.

**Methods:**

The study included 66 patients diagnosed with ADHD and 70 healthy adolescents who were similar to the case group in terms of age and gender. Clinical evaluation of the case and control groups were performed with "The Schedule for Affective Disorders and Schizophrenia for School-Age Children-Present and Lifetime Version, DSM-5 November 2016-Turkish Adaptation (K-SADS-PL-DSM-5-T)". "Conners-Wells Adolescent Self-Report Scale - Revised Short Form (CASS-RS)" and "Set of Early Maladaptive Schema Questionnaires for Children and Adolescents between the ages of 10-16" were administered to all participants.

**Results:**

It was determined that the schema scores of "dependence/incompetence", "vulnerability to harm or illness", "entitlement/grandiosity", "insufficient self-control", "subjugation" were significantly higher in the ADHD group than in the control group. A negative, significant relationship was found between age and "enmeshment/undeveloped self", "entitlement/grandiosity" and "insufficient self-control" schema scores. There was no significant difference between schema scores in terms of gender in the ADHD group. Additionally, no significant relationship was found between the education level of the parents, family income levels and schema scores. A significant positive relationship was found between the CASS-RS scores and all schema scores. As a result of the regression analysis, it was determined that CASS-RS scores positively predicted all schema scores, while the age variable negatively predicted only the schema scores of "enmeshment/undeveloped self".

**Discussion:**

Results of this study showed that there were significant differences in adolescents in the ADHD group compared to the control group in terms of early maladaptive schemas, and that ADHD symptom levels was associated with early maladaptive schemas.

## 1 Introduction

Attention Deficit Hyperactivity Disorder (ADHD) is one of the most common psychiatric disorders in children and adolescents. In the DSM-5, it is defined as a neurodevelopmental disorder characterized by decreased sustained attention, increased impulsivity, and/or mobility (1). It is known that the average prevalence in children and adolescents is 5.8-7.2% (2). ADHD has many lifelong negative effects, including early childhood.

ADHD is a disease whose clinical findings change with age and causes social, academic and mental deterioration (3). In the preschool period, difficulties in movement and impulse control and emotion regulation are at the forefront, and attention deficit and related academic difficulties are added to the existing findings in the school period. Although there is a decrease in hyperactivity with age, attention deficit and organizational difficulties continue (4). During adolescence, behavioral problems due to impulsivity and emotion regulation difficulties, alcohol substance use, risky sexual behaviors, concomitant depression and anxiety disorders can be seen (5). These findings cause children and adolescents to have difficulties in family and friend relationships, in the school environment, and to accumulate negative life experiences in almost every field. According to theorists, negative life events experienced early in life can affect a person's cognitive structure and cause them to develop maladaptive schemas (6, 7).

Early maladaptive schemas are defined as stable and change-resistant patterns consisting of memories, thoughts, and emotions that develop in line with a person's temperament and life experiences (8). Maladaptive schemas that are formed in childhood and adolescence and persist throughout life can become active in stressful situations, causing the person to experience negative emotions and deteriorate their functionality. According to Young et al. (8), failure to meet basic emotional needs in childhood and repetitive negative experiences lead to the development of maladaptive schemas in the early period. According to Young (8), the main source of psychopathology is early maladaptive schemas, and in this direction, he developed the schema-focused therapy model.

Schema therapy is a holistic therapy method in which traditional cognitive behavioral treatments and concepts are developed, including attachment theories and psychoanalysis. The aim of schema therapy is to gain insight into the person's dysfunctional schemas and their origin, to understand their emotional needs that were not met during childhood, and to reach the competence to meet their basic emotional needs in daily life (6, 8). The effectiveness of schema therapy, which was developed for the treatment of Axis I disorders and personality disorders that do not benefit from cognitive behavioral therapy, has been shown in many studies (9, 10).

Studies show that early maladaptive schemas are associated with many psychiatric disorders such as depression, anxiety disorder, obsessive-compulsive disorder, post-traumatic stress disorder, substance use disorder, and eating disorders (11–15). Research shows that early maladaptive schemas are also negatively affected in ADHD (16). In a cross-sectional study conducted by Philipsen et al. (17) with adult ADHD patients, it was determined that almost all schema maladaptive schemas were significantly higher in ADHD patients compared to the control group, and especially the schemas of “failure”, “defectiveness/shame”, “subjugation” and “emotional deprivation” were the most significantly impaired areas in adult ADHD patients. A similar study was conducted by Kiraz and Sertçelik (18), and it was determined that all early maladaptive schemas were significantly higher in adult ADHD patients, and the most affected schema areas were “failure,” “emotional inhibition,” “insufficient self-control” and “social isolation”.

When the literature on the subject is examined, it is seen that the existing studies are mostly conducted with adult patients (17–20), and the early maladaptive studies in children and adolescents with ADHD are quite limited (21, 22). The periods when the clinical findings of ADHD are most evident are childhood and adolescence (3, 4). Considering that early maladaptive schemas develop early in life, it is thought that the evaluation of the adolescent age group with ADHD in terms of maladaptive schemas is important in terms of psychological adjustment, comorbid psychiatric diseases and their treatment. In this study, it was aimed to evaluate early maladaptive schemas in adolescents diagnosed with ADHD and to examine the relationship between ADHD symptom severity and maladaptive schema.

## 2 Materials and methods

### 2.1 Participants

The case group of the study was selected from among adolescents aged 12-16 years who applied to Sivas Cumhuriyet University Faculty of Medicine, Department of Child and Adolescent Psychiatry between 22.09.2023 and 15.12.2023. The case group consists of 66 adolescents who were diagnosed with ADHD according to DSM-5 diagnostic criteria as a result of psychiatric evaluation by a child and adolescent psychiatrist. It was determined that 29 (44%) of the ADHD patients included in the study were diagnosed for the first time, and 37 (56%) of the cases had received only medication treatment for a certain period of time with the diagnosis of ADHD in the past, but were not currently receiving any treatment. In the control group, 70 healthy adolescents from a secondary school were randomly selected from a secondary school and whose age and gender were similar to the case group. The sample size was calculated by G*Power (3.1.9.4) analysis based on the nominal significance level of α=0.05, the medium effect size of r=0.5 and the power value of 1-β=0.8, and the minimum total sample size (n) was determined as 128 as a result of the evaluation. Those younger than 12 years of age and older than 16 years of age, those with psychiatric disorders other than ADHD, intellectual disability, autism, psychotic disorders, and any other chronic medical disorders were not included in the study.

After ethics committee approval is received the adolescents and their parents included in the study were informed about the study and their written consent was obtained. Clinical evaluation of the case and control groups were performed with the Schedule for Affective Disorders and Schizophrenia for School-Age Children-Present and Lifetime Version, DSM-5 November 2016-Turkish Adaptation. Conners-Wells Adolescent Self-Report Scale - Revised Short Form and Set of Early Maladaptive Schema Questionnaires for Children and Adolescents between the ages of 10-16 were applied to all participants.

### 2.2 Clinical assessment and data collection tools

#### 2.2.1 The schedule for affective disorders and schizophrenia for school-age children-present and lifetime version, DSM-5 November 2016-Turkish Adaptation (K-SADS-PL-DSM-5-T)

Psychiatric evaluations of the participants in the case and control groups were conducted by a child and adolescent psychiatrist through a semi-structured psychiatric interview K-SADS-PL-DSM-5-T was carried out. Kauffman et al. (23) this evaluation tool, developed by, consists of three parts. The first part consists of an unstructured initial interview, the second part consists of a diagnostic screening interview, and the third part consists of a general evaluation scale for children. Turkish validity and reliability studies by Ünal et al. (24) conducted by.

#### 2.2.2 Sociodemographic data form

In this form prepared by the researchers, information such as the age of the participant, gender, parental age, education level, family income level is questioned. The researchers filled out the parameters of this form during interviews with participants and their parents.

#### 2.2.3 Conners-Wells Adolescent Self-Report Scale - Revised Short Form

This scale, developed by Conners et al. (25), is a self-report scale applied to adolescents between the ages of 12-17 that evaluates ADHD symptoms in adolescents. The 4-point Likert-type scale, which consists of a total of 27 items, has three sub-dimensions: “conduct disorder”, “cognitive problems-inattention”, “hyperactivity”, and the “ADHD index”, which is an auxiliary scale that evaluates children and adolescents at risk of ADHD. The high scores obtained from the scale indicate that the adolescent has a high severity of the problem defined by the scale. In this study, the severity of ADHD symptoms of the participants was evaluated with the total scores of CASS-RS. The Turkish validity and reliability study of the scale was conducted by Kaner et al. (26).

#### 2.2.4 Set of early maladaptive schema questionnaires for children and adolescents between the ages of 10–16

This scale, developed by Guner (27), is a 5-point Likert-type self-report scale consisting of 97 items in total. The scale has 5 different schema domains [(1) disconnection and rejection, (2) impaired autonomy and performance, (3) impaired limits, (4) other directedness, (5) over vigilance and inhibition)] and sub-schema scales consisting of 15 factors. The subscales in schema domains are, respectively, (1) “defectiveness/shame”, “mistrust/abuse”, “emotional deprivation”, “abandonment/instability” within the schema domain for disconnection and rejection, (2) “failure”, “dependence/incompetence”, “enmeshment/undeveloped self”, “vulnerability to harm or illness” within the schema domain of impaired autonomy and performance, (3) “entitlement/grandiosity”, “insufficient self-control” within the domain of impaired limits, (4) “subjugation”, “self-disapproval”, “approval-seeking” within the domain of other directedness, (5) “pessimism” and “punitiveness” within the schema domain of over vigilance and inhibition. The high scores obtained from the subscales indicate that the relevant schema level is high.

### 2.3 Statistical analysis

All data were evaluated using Statistical Package for Social Science (SPSS) Windows version 24.0 software. For the analysis of the conformity of the data to the normal distribution; the “Kolmogorov-Smirnov test” and “Levene's test” were used for the conformity of the homogeneous variance assumption. While comparing ADHD and the control group in terms of sociodemographic characteristics, “Chi-square test” was applied for categorical variables and “Independent sample T test” was applied for continuous variables. “Independent sample T test” was used to compare the scores of the groups on the CASS-RS and the Set of Early Maladaptive Schema Questionnaires for Children and Adolescents between the ages of 10-16. “Independent sample T test” was used to evaluate the shema scores in terms of gender. The relationships of continuous variables were analyzed with the “Pearson correlation test”, and the relationships of categorical variables were analyzed with the “Spearman correlation test”. The predictive effect of age and CASS-RS scores on the shema scores was tested with “Multiple linear regression analysis”. The p-value for statistical significance was accepted as <0.05.

## 3 Results

The sociodemographic characteristics of the ADHD and control group are shown in **Table 1**. There was no significant difference between the groups in terms of age, gender, age of parents, education level of parents, family type, and family income level of adolescents (p> 0.05) (**Table 1**).

**Table 1:** Sociodemographic characteristics of the ADHD group and the control group.

		ADHD Group		Control Group		p
		N	%	N	%	
Age (Mean ± SD)		13.74 ± 0.82		13.28 ± 0.45		0.32^b^
Gender	Female	21	31.8	25	35.7	0.23^a^
	Male	45	68.2	45	64.3	
Mother's age (Mean ± SD)		39.93 ± 5.23		40.77 ± 4.57		0.32^b^
Father's age (Mean ± SD)		44.31 ± 5.65		44.60 ± 6.23		0.78^b^
Mother's Education Level						
	Primary Education	30	45.5	22	31.4	0.34^a^
	High School	28	42.3	33	47.2	
	University	8	12.2	15	21.4	
Father's Education Level						
	Primary Education	22	33.3	17	24.2	0.29^a^
	High School	30	45.5	36	51.4	
	University	14	21.2	17	24.2	
Family Type						
	Nuclear Family	52	78.8	51	72.8	0.97^a^
	Extended family	6	9.1	5	7.2	
	Separated Parents	5	7.6	9	12.8	
	Single Parent	3	4.5	5	7.2	
Family Income Level						
	Good	18	27.3	25	35.7	0.56^a^
	Middle	45	68.2	42	60.0	
	Bad	3	4.5	3	4.30	

^a^Chi-square Test, ^b^Independent Sample T – Test.

When the CASS-RS scores of the adolescents in the ADHD and control groups were evaluated, it was determined that the mean CASS-RS scores of the adolescents in the ADHD group were 31.78 ± 13.70, and the control group was 21.00 ± 8.26, and the CASS-RS scores of the adolescents in the ADHD group were significantly higher than the control group (p < 0.001). When the groups were evaluated in terms of the scores of the Set of Early Maladaptive Schema Questionnaires for Children and Adolescents between the ages of 10-16, it was found that the schema scores of “dependency/incompetence” (p = 0.01), “vulnerability to harm or illness” (p = 0.04), “entitlement/grandiosity” (p = 0.02), “insufficient self-control” (p < 0.001), “subjugation” (p<0.001) were significantly higher in the ADHD group than in the control group (**Table 2**).

**Table 2:** Comparison of the scores of the ADHD group and the control group with CASS-RS and the Set of Early Maladaptive Schema Questionnaires for Children and Adolescents between the ages of 10-16.

	ADHD Group (N=66) (Ort. ± SS)	Control Group (N=70) (Ort. ± SS)	p
**CASS-RS**	31.78 ± 13.70	21.00 ± 8.26	0.000**
**Defectiveness/shame**	11.72 ± 4.29	11.38 ± 4,39	0.64
**Mistrust/abuse**	16.07 ± 6,66	17.02 ± 6.58	0.40
**Emotional deprivation**	21.04 ± 9.76	20.37 ± 8.21	0.50
**Abandonment/instability**	9.56 ± 3.98	9.30 ± 4.01	0.70
**Failure**	17.60 ± 6.18	16.74 ± 7.44	0.46
**Dependence/incompetence**	16.66 ± 6.76	13.71 ± 6.40	0.01*
**Enmeshment/undeveloped self**	8.51 ± 3.62	8.05 ± 3.61	0.46
**Vulnerability to harm or illness**	8.65 ± 3.18	7.51 ± 3.46	0.04*
**Entitlement/grandiosity**	39.90 ± 14.86	34.57 ± 10.78	0.02*
**Insufficient self-control**	16.00 ± 5.19	12.54 ± 4.70	0.000**
**Subjugation**	16.40 ± 6.86	13.42 ± 4.98	0.000**
**Self-disapproval**	9.77 ± 4.52	10.62 ± 5.21	0.31
**Approval-seeking**	10.77 ± 4.85	9.45 ± 4.22	0.09
**Pessimism**	20.74 ± 8.13	22.74 ± 8.24	0.15
**Punitiveness**	12.34 ± 4.59	11.88 ± 4.08	0.53

**p < 0.001, *p < 0.05; Independent Sample T - Test; CASS-RS, Conners-Wells Adolescent Self-Report Scale – Revised Short Form.

When the relationship between the ages of the adolescents in the ADHD group and the scores of Set of Early Maladaptive Schema Questionnaires for Children and Adolescents between the ages of 10-16 was evaluated, a significant correlation was found between age and “enmeshment/undeveloped self” (p = 0.004), “entitlement/grandiosity” (p = 0.01), and "insufficient self-control" at a negative weak level (p = 0.003) at a negative weak level. There was no significant relationship between the other twelve schema scores and the age variable (p> 0.05). Additionally, no significant relationship was found between the education level of the parents, family income levels and schema scores (p > 0.05) (**Table 3**).

**Table 3:** The relationship between the sociodemographic characteristics and CASS-RS scores with the scores of Set of Early Maladaptive Schema Questionnaires for Children and Adolescents between the ages of 10-16.

		Age of adolescents	Mothers' education	Fathers' education	Family income	CASS-RS
Defectiveness/shame	r	0.00	0.10	0.04	-0.11	0.53
	p	0.96	0.38	0.72	0.37	0.000**
Mistrust/abuse	r	-0.16	0.01	-0,13	-0,02	0.43
	p	0.19	0.93	0,28	0,81	0.000**
Emotional deprivation	r	0.47	0.09	0.04	0.08	0.42
	p	0.70	0.46	0.74	0.51	0.000**
Abandonment/instability	r	0.12	0.11	-0.64	0.11	0.43
	p	0.19	0.83	0.27	0.18	0.000**
Failure	r	-0.12	-0.04	-0.08	-0.19	0.50
	p	0.31	0.97	0.48	0.12	0.000**
Dependence/incompetence	r	-0.06	0.09	-0.08	-0.16	0.40
	p	0.61	0.46	0.51	0.17	0.001*
Enmeshment/undeveloped self	r	-0.34	-0.07	-0.09	-0.03	0.43
	p	0.004*	0.53	0.45	0.76	0.000**
Vulnerability to harm or illness	r	-0.17	0.03	-0.04	-0.07	0.47
	p	0.15	0.81	0.73	0.56	0.000**
Entitlement/grandiosity	r	-0,31	0.40	-0.17	-0.18	0.58
	p	0.01*	0.75	0.15	0.14	0.000**
Insufficient self-control	r	-0.25	0.15	0.00	-0.25	0.56
	p	0.003*	0.20	0.94	0.45	0.000**
Subjugation	r	0.10	0.01	0.07	0.04	0.38
	p	0.66	0.93	0.55	0.72	0.001*
Self-disapproval	r	-0.00	0.05	-0.09	-0.15	0,37
	p	0.98	0.64	0.43	0.21	0.01*
Approval-seeking	r	-0.11	0.05	-0.46	-0.22	0.45
	p	0.34	0.68	0.71	0.06	0.000**
Pessimism	r	-0.11	0.28	0.00	-0.15	0.51
	p	0.36	0.07	0.98	0.21	0.001*
Punitiveness	r	-0.18	0.00	-0.04	0.07	0.39
	p	0.13	0.96	0.72	0.57	0.000**

*p<0.05, **p<0.001; Pearson Correlation Test, Spearman Correlation Test; CASS-RS, Conners-Wells Adolescent Self-Report Scale - Revised Short Form.

When the relationship between the CASS-RS in the ADHD group and the scores of Set of Early Maladaptive Schema Questionnaires for Children and Adolescents between the ages of 10-16 was evaluated, a significant positive correlation was found between the CASS-RS and all scheme scores. While there was a weak relationship between CASS-RS and “self-disapproval” (p = 0.01), “subjugation” (p = 0.001) schemas, a moderately significant relationship was found with the other 13 schema scores. (**Table 3**).

There was no significant difference between male and female adolescents in the ADHD group in terms of the scores of the Early Maladaptive Schema Scale Set for Children and Adolescents aged 10-16 years (Independent sample T test; p > 0.05).

Multiple linear regression models were established to evaluate the relationship between the scores of the Set of Early Maladaptive Schema Questionnaires for Children and Adolescents between the ages of 10-16 with age and CASS-RS scores in the ADHD group. In the regression model established for 15 different schema scores, it was found that the CASS-RS scores were “defectiveness/shame” (β = 0.18, t = 5.52, p < 0.001), “mistrust/abuse” (β = 0.23, t = 4.06, p < 0.001), “emotional deprivation” (β = 0.34, t = 4.14, p < 0.001), “abandonment/instability” (β = 0.12, t = 3.62, p = 0.001), “failure” (β = 0.22, t = 4.44, p < 0.001), “dependence/incompetence” (β = 0.21, t = 3.57, p = 0.001), “enmeshment/undeveloped self” (β = 0.09, t = 3.10, p = 0.003), “vulnerability to harm or illness” (β = 0.11, t = 4.03, p < 0.001) “entitlement/grandiosity” (β = 0.58, t = 5.07, p < 0.001), “insufficient self-control” (β = 0.20, t = 4.93, p < 0.001), “subjugation” (β = 0.20, t = 3.31, p = 0.002), “self-disapproval” (β = 0.11, t = 2.69, p = 0.009), “approval-seeking” (β = 0.16, t = 3.95, p < 0.001), “pessimism” (β = 0.31, t = 4.61, p < 0.001), “punitiveness” (β = 0.12, t = 3.09, p = 0.003) had a positive effect on schema scores. In addition, a significant negative correlation was found between the age variable and the schema scores of “enmeshment/undeveloped self” (β = -0.49, t = -2.06, p = 0.04) (**Table 4**).

**Table 4:** Regression analysis of the set of early maladaptive schema questionnaires for children and adolescents between the ages of 10-16 scores, age and CASS-RS scores.

Dependent variable	Independent variable	F	R^2^	Beta	t	p
Defectiveness/shame	Age	10.04	0.32	0.53	1.91	0.06
	CASS-RS			0.18	5.52	0.000**
Mistrust/abuse	Age	7.30	0.26	0.33	0.74	0.46
	CASS-RS			0.23	4.06	0.000**
Emotional deprivation	Age	5.94	0.22	0.95	1.39	0.16
	CASS-RS			0.34	4.14	0.000**
Abandonment/instability	Age	5.33	0.20	-0.14	-0.50	0.61
	CASS-RS			0.12	3.62	0.001*
Failure	Age	7.06	0.25	0.02	0.06	0.94
	CASS-RS			0.22	4.44	0.000**
Dependence/incompetence	Age	4.94	0.19	0.10	0.20	0.83
	CASS-RS			0.21	3.57	0.001*
Enmeshment/undeveloped self	Age	6.88	0.25	-0.49	-2.06	0.04*
	CASS-RS			0.09	3.10	0.003*
Vulnerability to harm or illness	Age	6.50	0.23	-0.12	-0.52	0.60
	CASS-RS			0.11	4.03	0.000**
Entitlement/grandiosity	Age	13.04	0.38	-1.68	-1.82	0.73
	CASS-RS			0.58	5.02	0.000**
Insufficient self-control	Age	10.83	0.34	-0.39	-1.18	0.24
	CASS-RS			0.20	4.93	0.000**
Subjugation	Age	3.99	0.16	0.15	0.30	0.76
	CASS-RS			0.20	3.31	0.002*
Self-disapproval	Age	2.84	0.12	0.16	0.48	0.62
	CASS-RS			0.11	2.69	0.009*
Approval-seeking	Age	5.56	0.21	0.02	0.06	0.95
	CASS-RS			0.16	3.95	0.000**
Pessimism	Age	7.38	0.26	0.24	0.44	0.66
	CASS-RS			0.31	4.61	0.000**
Punitiveness	Age	3.99	0.16	-0.18	-0.54	0.58
	CASS-RS			0.12	3.09	0.003*

*p < 0.05, **p < 0.001; Multiple Linear Regression Analysis; CASS-RS: Conners-Wells Adolescent Self-Report Scale - Revised Short Form.

## 4 Discussion

In the results of our study, it was determined that the schema scores of “dependency/incompetence”, “vulnerability to harm or illness”, “entitlement/grandiosity”, “insufficient self-control”, “subjugation” were significantly higher in the case group diagnosed with ADHD than in the control group. When the relationship between the age levels of the adolescents in the ADHD group and the early maladaptive schema scores was evaluated, a negative weak correlation was found between age and the schema scores of “enmeshment/undeveloped self”, “entitlement/grandiosity”, and “insufficient self-controls”. In the ADHD group, there was no significant difference between the early maladaptive schema scores in male and female adolescents. Additionally, no significant relationship was found between the education level of the parents, family income levels and schema scores. While there was a weakly relationship between ADHD symptom levels and “self-disapproval”, “subjugation” schemas, a moderately significant relationship was found with the other 13 schema scores. As a result of the regression analysis, it was determined that ADHD symptom levels positively predicted all schema scores, while the age variable negatively predicted only the schema scores of "enmeshment/undeveloped self"

The "dependency/incompetence" schema refers to the inability of the person to take responsibility for life, to make and implement decisions on their own, and to set the right goals. Individuals with this schema think that they cannot act independently of their families, so they have difficulty in forming an identity and establishing an independent life (8). In our study, dependency/incompetence schema levels were found to be significantly higher in adolescents in the ADHD group than in the control group. One of the main features of ADHD is impairment in executive functions (28). Limitations in attention, working memory, and planning skills cause difficulties in fulfilling daily routines and in social and academic areas (29). With the effect of difficulties and negative experiences in these areas from the early period of life, thoughts of inadequacy and dependency in areas directly related to executive functions such as taking and fulfilling responsibilities and setting correct goals are expected during adolescence.

Individuals with a “vulnerability to harm or illness” schema have a constant belief that they will face a disaster that they cannot cope with. They have an exaggerated and unrealistic expectation about an external disaster such as a physical, mental and/or natural disaster. Young et al. (8) suggested that schemas associated with the theme of danger are associated with traumatic experiences (abuse and neglect) in childhood. Studies show that individuals with ADHD are exposed to negative life events as of childhood (30, 31). In a case-control study conducted by Gokten et al. (32) with 104 children with ADHD, it was determined that children with ADHD were exposed to more physical and emotional abuse, physical and emotional neglect than the control group. When our results are evaluated in the light of the literature, we think that the schema of vulnerability to harm or illness may be affected in adolescents with ADHD. In our study, adolescents' negative life experiences and childhood traumas were not questioned. In order to better interpret our results, longitudinal follow-up studies are needed to evaluate the relationship between early maladaptive schemas of children with ADHD and childhood traumas.

In our study, it was determined that the levels of both early maladaptive schemas, namely “entitlement/grandiosity” and “insufficient self-control”, which were categorized within the “impaired limits domain”, were significantly higher in the ADHD group than in the control group. Both schemas are related to self-discipline, the inability to set internal boundaries for oneself and others (8). Impulsivity and inadequate self-control are core symptoms of ADHD (3, 4). Considering that impulsivity increases during adolescence, it is expected that adolescents with ADHD have difficulty in areas where self-control is required and disruption of schema areas related to boundaries in behavior and relationships. Individuals with entitlement/grandiosity schemes are individuals who do not recognize boundaries and rules in relationships, tend to see themselves as right in every situation, and have weak empathy skills. Studies have shown that this schema is associated with narcissistic personality disorder (33, 34). In our study, adolescents with ADHD were not evaluated for personality disorder. Although our results suggest that the impaired limits schema domain may be affected in adolescents with ADHD, there is a need for studies evaluating the relationship between personality patterns and early maladaptive schemas in ADHD in order to better interpret our findings.

In our study, the “subjugation” schema level of the ADHD group was found to be significantly higher than the control group. The subjugation is the relationship with thinking that one's own ideas, feelings, and needs are unimportant in one's relationship with others. These individuals, who tend to suppress their emotions and/or needs, may experience passive-aggressive behaviors, avoidance of emotional intimacy, and outbursts of anger in their social relationships. The origin of the subjugation schema is associated with overly punitive parental attitudes (8). Children and adolescents with ADHD are defined as “difficult children” depending on the difficulties experienced in the care process, and they may be exposed to extremely authoritarian/punitive attitudes by their parents during the upbringing process (35, 36). In the study conducted by Shahinuzzaman et al. (37) with 386 parents, a significant relationship was found between children's ADHD findings and authoritarian parental attitudes. In a cross-sectional study conducted by Iqbal and Anis-ul-Haque (21) with 100 adolescents diagnosed with ADHD, a positive correlation was found between negative parental attitudes and the total scores of early maladaptive schemas. Our results appear to be consistent with the literature. However, parental attitudes were not evaluated in our study. In order to better interpret our results, it is thought that there is a need for studies examining the relationship between parental attitudes and early maladaptive schemas in children and adolescents with ADHD and the effects of negative parental attitudes on maladaptive schemas.

When the studies on the subject are examined, it is seen that the studies examining the early maladaptive schemas in ADHD are mostly carried out with the adult patient group (16–20). In a study conducted by Philipsen et al. (17) with 78 adult ADHD patients, it was found that all schema levels were significantly higher in ADHD patients than in the control group, except for vulnerability to harm or illness, enmeshment/undeveloped self, self-sacrifice, insufficient self-control and approval-seeking. In a study conducted by Kiraz and Sertcelik (18) with 55 adult ADHD patients and 52 control groups, similarly, all schema levels were found to be significantly higher in the ADHD group, and failure, emotional inhibition, insufficient self-control, and social isolation schemas were determined as the most associated schemas with ADHD. In our study, there was no significant difference between the groups in terms of other schemas other than “dependency/incompetence”, “vulnerability to harm or illness”, “entitlement/grandiosity”, “insufficient self-control”, “subjugation” schema scores. In Yilmaz Turkel's (22) unpublished thesis study with 50 children with ADHD, the results were similar to our study, and there was no significant difference between the ADHD group and the control group in terms of other schemas other than emotional deprivation and mistrust/abuse. Our results are partially consistent with the literature. In our study, the reason for the difference between the case and control group in fewer incompatible schemes compared to the studies with adults may be related to the average age of the research sample. Adolescence can be an early age period for maladaptive schemas to form and establish early compared to adults. Our inconsistent results with the studies conducted in adult ADHD patients can also be explained by the fact that most of the adolescents in the ADHD group of our study [37 (56%)] were diagnosed and treated in the past. Early control of ADHD symptoms may support individuals to have fewer negative experiences and therefore fewer maladaptive schemas.

As a result of our study, it was determined that there was a positive relationship between ADHD symptom levels and schema scores, and that ADHD severity positively predicted all schema levels. Research results show that ADHD symptom severity is associated with lifelong negative experiences (30, 31, 38). Although our results suggest that early maladaptive schemas are associated with the symptoms of ADHD, it is thought that our findings can be better interpreted with future studies evaluating negative life experiences and traumatic experiences in children and adolescents with ADHD.

In our study, a negative significant relationship was found between the age variable and the “enmeshment/undeveloped self”, “entitlement/grandiosity”, “insufficient self-control” schemas in the ADHD group, and it was found that age had a negative effect on the “enmeshment/undeveloped self” schema. In terms of gender, there was no significant difference in early incompatible schemes. The results of studies examining the relationship between genealogical characteristics and early maladaptive schemas in ADHD vary. In the study of Iqbal and Anis-ul-Haque (21), it was found that the levels of early maladaptive schemas increased significantly with age, while self-sacrifice schemas were high in female adolescents and insufficient self-control, emotional deprivation, emotional inhibition, defectiveness/shame, vulnerability to harm or illness, and entitlement/grandiosity schemas were high in boys. In the study of Yilmaz Turkel (22), insufficient self-control schema was found to be significantly higher in male adolescents with ADHD than in girls, and no significant difference was found in terms of gender at other schema levels. When our results are evaluated in the light of the literature, it suggests that different variables may play a role in the relationship between age and gender with early maladaptive schemas in adolescents with ADHD.

In Yilmaz Turkel's (22) study, schema levels of failure, entitlement/grandiosity, insufficient self-control and subjugation were found to be high in those with low father education level, while no difference was found between the schemas according to the mother's education level. In our study, no significant relationship was found between the education levels of the mothers and fathers, as well as family income levels and schema levels. Although our results seem partially compatible with the literature, our findings will be more meaningful with studies with large samples on the subject.

### 4.1 Limitations

There are some limitations of our study. The small size of the sample and the fact that the sample includes patients in a certain age range (12-16 years) is an important limitation. According to the schema therapy approach, the most important factors in the formation of early maladaptive schemas are the person's temperament, negative life experiences, and unmet needs in relation to the care data (6, 8). In our study, the participants' temperament, childhood traumatic experiences, attachment characteristics, and parental attitudes were not evaluated. Another limitation of our study is that most of the patients in the case group (56%) were diagnosed with ADHD in the past and treated for a certain period of time. Cases that received treatment in the past were mostly followed up in a university hospital, were only treated with medication, and did not receive any therapy support. However, when evaluating our results, it is thought that it should be taken into consideration that some of the ADHD cases had received medication in the past and that this situation may have affected early maladaptive schemas. It is thought that longitudinal studies with large samples including children and adolescents of different ages who were diagnosed for the first time are needed to determine the early maladaptive schemas in ADHD and to examine the causal relationship of the schemas with the clinical findings of the disease in detail. In addition, there is a need for research to evaluate the mediating effects of factors known to be associated with early maladaptive schemas such as children's and adolescents' temperament, personality patterns, attachment characteristics, parental attitudes, and negative life experiences.

### 4.2 Conclusion

Our results showed that there were significant differences in adolescents in the ADHD group compared to the control group in terms of early maladaptive schemas, and that ADHD symptom levels was associated with early maladaptive schemas. Although our findings suggest that early schemas may be affected in adolescents with ADHD, it is thought that the interpretability of our results will increase with future research designed to address the limitations of the subject. This study is one of the few studies examining early maladaptive schemas with ADHD (21, 22). It is known that early schemas are closely related to psychological adjustment and the etiology of many diseases such as depression and anxiety disorder (10, 11, 16, 39). When our results are evaluated in the light of the literature, it is thought that considering early maladaptive schemas in the treatment approach of adolescents with ADHD is important both in terms of supporting current psychological adjustment and protecting them from comorbid psychiatric disorders with a high risk of developing in the future.

## Data Availability

The raw data supporting the conclusions of this article will be made available by the authors, without undue reservation.

## References

[1] American Psychiatric Association. Diagnostic statistical manual of mental disorder. 5th ed. Wahington, DC: American Psychiatric Association (2013).

[2] Willcutt EG. The prevalence of DSM-IV attention-deficit/hyperactivity disorder: a meta-analytic review. Neurotherapeutics. (2012) 9:490–9. doi: 10.1007/s13311-012-0135-8 PMC3441936 22976615

[3] Schachar R Tannock R. Syndromes of Hyperactivity and Attention Deficit. In: Rutter M Taylor E, editors. Child and Adolescent Psychiatry Textbook. Blackwell Science, Oxford (2002).

[4] Moriyama T Cho A Verin R Fuentes J Polanczyk GV. Attention deficit hyperactivity disorder. In: Rey JM, editor. IACAPAP E-Textbook of Child and Adolescent Mental Health. International Association for Child and Adolescent Psychiatry and Allied Professions, Geneva (2012).

[5] Lee SS Humphreys KL Flory K Liu R Glass K. Prospective association of childhood attention-deficit/hyperactivity disorder (ADHD) and substance use and abuse/dependence: A meta-analytic review. Clin Psychol Rev. (2011) 31:328–41. doi: 10.1016/j.cpr.2011.01.006 PMC3180912 21382538

[6] Young JE. Cognitive therapy for personality disorders: A schema-focused approach. Florida: Professional Resource Press/Professional Resource Exchange (1994).

[7] Nordahl HM Holthe H Haugum JA. Early maladaptive schemas in patients with or without personality disorders: Does schema modification predict symptomatic relief? Clin Psychol Psychother. (2005) 12:142–9. doi: 10.1002/cpp.430

[8] Young JE Klosko JS Weishaar ME. Schema Therapy: A Practitioner’s Guide. New York: The Guilford Press (2003).

[9] Barazandeh H Kissane DW Saeedi N Gordon M. A systematic review of the relationship between early maladaptive schemas and borderline personality disorder/traits. Pers Individ Dif. (2016) 94:130–9. doi: 10.1016/j.paid.2016.01.021

[10] Thimm JC Chang M. Early maladaptive schemas and mental disorders in adulthood: A systematic review and meta-analysis. Int J Cognit Ther. (2022) 15:371–413. doi: 10.1007/s41811-022-00149-7

[11] Tariq A Reid C Chan SW. A meta-analysis of the relationship between early maladaptive schemas and depression in adolescence and young adulthood. Psychol Med. (2021) 51:1233–48. doi: 10.1017/S0033291721001458 34109934

[12] Calvete E Orue I Hankin BL. Early maladaptive schemas and social anxiety in adolescents: The mediating role of anxious automatic thoughts. J Anxiety Disord. (2013) 27:278–88. doi: 10.1016/j.janxdis.2013.02.011 23602941

[13] Jaeger T Moulding R Yang YH David J Knight T Norberg MM. A systematic review of obsessive-compulsive disorder and self: Self-esteem, feared self, self-ambivalence, egodystonicity, early maladaptive schemas, and self-concealment. J Obsessive Compuls Relat Disord. (2021) 31:100665. doi: 10.1016/j.jocrd.2021.100665

[14] Cockram DM Drummond PD Lee CW. Role and treatment of early maladaptive schemas in Vietnam veterans with PTSD. Clin Psychol Psychother. (2010) 17:165–82. doi: 10.1002/cpp.690 20486158

[15] Zhu H Luo X Cai T He J Lu Y Wu S. Life event stress and binge eating among adolescents: The roles of early maladaptive schemas and impulsivity. Stress Health. (2016) 32:395–401. doi: 10.1002/smi.2634 25688978

[16] Bär A Bär HE Rijkeboer MM Lobbestael J. Early Maladaptive Schemas and Schema Modes in clinical disorders: A systematic review. Psychol Psychother-T. (2023) 96:716–47. doi: 10.1111/papt.12465 37026578

[17] Philipsen A Lam AP Breit S Lücke C Müller HH Matthies S. Early maladaptive schemas in adult patients with attention deficit hyperactivity disorder. Atten Defic Hyperact Disord. (2017) 9:101–11. doi: 10.1007/s12402-016-0211-8 28012033

[18] Kiraz S Sertcelik S. Adult attention deficit hyperactivity disorder and early maladaptive schemas. Clin Psychol Psychother. (2021) 28:1055–64. doi: 10.1002/cpp.2569 33586830

[19] Miklósi M Máté O Somogyi K Szabó M. Adult attention deficit hyperactivity disorder symptoms, perceived stress, and well-being: the role of early maladaptive schemata. J Nerv Ment Dis. (2016) 204:364–9. doi: 10.1097/NMD.0000000000000472 26825377

[20] Rahmani N Talaei M Hadianfard H. Comparison of maladaptive schemas, metacognitive and meta-emotional components in individuals with attention-deficit/hyperactivity disorder and healthy individuals. J Psychol New Ideas. (2023) 15:1–13.

[21] Iqbal R Anis-ul-Haque M. Impact of parenting practices and family functioning on psychological adjustment of adolescents with ADHD: role of early maladaptive schemas. Int J Educ Psychol. (2023) 2:27–52. doi: 10.58425/ijpce.v2i2.221

[22] Yilmaz Turkel G. Investigation of the existence of early maladaptive schemas in children and adolescents diagnosed with attention deficit hyperactivity disorder and their relationship with parental attitudes. Kocaeli University Faculty of Medicine, Turkey (2019).

[23] Kaufman J Birmaher B Brent D Rao UMA Flynn C Moreci P. Schedule for affective disorders and schizophrenia for school-age children-present and lifetime version (K-SADS-PL): initial reliability and validity data. J Am Acad Child Adolesc Psychiatry. (1997) 36:980–8. doi: 10.1097/00004583-199707000-00021 9204677

[24] Ünal F Öktem F Çetin Çuhadaroğlu F Çengel Kültür ES Akdemir D Foto Özdemir D. Reliability and validity of the schedule for affective disorders and schizophrenia for school-age children-present and lifetime version, DSM-5 november 2016-turkish adaptation (K-SADS-PL-DSM-5-T). Turk Psikiyatri Derg. (2019) 30:42–50. doi: 10.5080/u23408 31170306

[25] Conners CK Wells KC Parker JDA Sitarenios G Diamond JM Powell JW. A new selfreport scale for assessment of adolescent psychopathology: Factor structure, reliability, validity and diagnostic sensitivity. J Abnorm Child Psychol. (1997) 25:487–97. doi: 10.1023/A:1022637815797 9468109

[26] Kaner S Buyukozturk S Iseri E Ak A Ozaydın L. Conners-wells' Adolescent self-report scale-long form: evaluation psychometric properties for turkish adolescents. Turkish J Clin Psychiatry. (2011) 14:71–84.

[27] Guner O. Validity, reliability and norm study of the Early Mismatch Schema Scales Set for 10-16 Years Old Children and Adolescents. Marmara University Institute of Educational Sciences, Istanbul (2013).

[28] Barkley RA. Behavioral inhibition, sustained attention, and executive functions: constructing a unifying theory of ADHD. Psychol Bull. (1997) 121:65. doi: 10.1037/0033-2909.121.1.65 9000892

[29] Willcutt EG Doyle AE Nigg JT Faraone SV Pennington BF. Validity of the executive function theory of Attention Deficit/Hyperactivity Disorder: A meta-analytic review. Biol Psychiatry. (2005) 57:1336–46. doi: 10.1016/j.biopsych.2005.02.006 15950006

[30] Brown NM Brown SN Briggs RD Germán M Belamarich PF Oyeku SO. Associations between adverse childhood experiences and ADHD diagnosis and severity. Acad Pediatr. (2017) 17:349–55. doi: 10.1016/j.acap.2016.08.013 28477799

[31] Craig SG Bondi BC O’Donnell KA Pepler DJ Weiss MD. ADHD and exposure to maltreatment in children and youth: A systematic review of the past 10 years. Curr Psychiatry Rep. (2020) 22:1–14. doi: 10.1007/s11920-020-01193-w 33161561

[32] Gokten ES Duman NS Soylu N Uzun ME. Effects of attention-deficit/hyperactivity disorder on child abuse and neglect. Child Abuse Negl. (2016) 62:1–9. doi: 10.1016/j.chiabu.2016.10.007 27770673

[33] Zeigler-Hill V Green BA Arnau RC Sisemore TB Myers EM. Trouble ahead, trouble behind: Narcissism and early maladaptive schemas. J Behav Ther Exp Psychiatry. (2011) 42:96–103. doi: 10.1016/j.jbtep.2010.07.004 20705282

[34] Soleimani E Lorzangeneh S. Investigating the relationship between early maladaptive schemas and narcissism in students. J Exp Psychopathol. (2021) 2:18–22. doi: 10.22098/JRP.2021.1201

[35] Corcoran J. The lived experience of parents of children with ADHD. In: Living with Mental Disorder. London: Routledge (2016). p. 15–103.

[36] Stevens AE Canu WH Lefler EK Hartung CM. Maternal parenting style and internalizing and ADHD symptoms in college students. J Child Fam Stud. (2019) 28:260–72. doi: 10.1007/s10826-018-1264-4

[37] Shahinuzzaman M Siddique N Hassan M Liza M Alia SS Nishad FR. Parenting practice, matrimonial instability, and children's Attention Deficit Hyperactivity Disorders. Int J Indian Psychol. (2022) 10:857–66. doi: 10.25215/1003.091

[38] Li T Franke B AriasVasquez A Mota NR. Mapping relationships between ADHD genetic liability, stressful life events, and ADHD symptoms in healthy adults. Am J Med Genet B Neuropsychiatr Genet. (2021) 186:242–50. doi: 10.1002/ajmg.b.32828 PMC8359274 33319511

[39] Gokce S Onal Sonmez A Yusufoglu C Yulaf Y Adak İ. The relationship between early maladaptive schemas and adolescent depressive disorder. Anadolu Psikiyatri Derg. (2017) 18:283–91. doi: 10.5455/apd.238486

